# Early age at menarche and metabolic cardiovascular risk factors: mediation by body composition in adulthood

**DOI:** 10.1038/s41598-020-80496-7

**Published:** 2021-01-08

**Authors:** Susana Bubach, Bernardo Lessa Horta, Helen Gonçalves, Maria Cecília Formoso Assunção

**Affiliations:** 1 Department of Health Sciences, Federal University of Espírito Santo, São Mateus, 29932-540 Brazil; 2 Post-Graduate Program in Epidemiology, Federal University of Pelotas, Pelotas, 29932-540 Brazil

**Keywords:** Disease prevention, Public health, Cardiovascular diseases, Cardiology, Diseases, Risk factors

## Abstract

Evidence suggests that early menarche increases cardiometabolic risk, and adiposity would be a possible mediator of this association. We assessed the association between age at menarche and metabolic cardiovascular risk factors and estimated the indirect effect of body composition in adulthood. In 1982, all hospital births in the city of Pelotas/Brazil, were identified and live births were examined and have been prospectively followed. At 30 years, information on age at menarche and metabolic cardiovascular risk factors was available for 1680 women. Mediation analysis was performed using G-computation to estimate the direct effect of age at menarche and the indirect effect of body composition. The prevalence of age at menarche < 12 years was 24.5% and was associated with higher mean diastolic blood pressure [β: 1.98; 95% CI: 0.56, 3.40], total cholesterol (β: 8.28; 95% CI: 2.67, 13.88), LDL-cholesterol (β: 6.53; 95% CI: 2.00, 11.07), triglycerides (β: 0.11; 95% CI: 0.03, 0.19). For diastolic blood pressure, total cholesterol, LDL-cholesterol, triglycerides, body composition assessed by fat mass index captured from 43.8 to 98.9% of the effect of early menarche, except to systolic blood pressure, HDL-cholesterol, C-reactive-protein. Suggesting that the effect of menarche age < 12 years on some metabolic cardiovascular risk factors is mediated partially by body composition in adulthood.

## Introduction

Cardiometabolic diseases are among the leading causes of death worldwide^1^, and deaths from cardiovascular diseases are estimated to increase from 17.5 million in 2012 to 22.2 million in 2030^2^. Regarding the factors associated with the development of cardiovascular diseases, modifiable factors include obesity, tobacco use, physical inactivity, excessive intake of salt and fat, alcohol abuse, unsatisfactory intake of fruits and vegetables, psychological stress, low socioeconomic status and inadequate access to health services^2^.

Evidence suggests that early menarche would also be associated with an increased risk of cardiometabolic events and mortality^3–9^, and the greater accumulation of fat in women with early age at menarche would be a possible mechanism to explain this association. Girls with early menarche (< 12 years) present a higher risk of obesity, and this association would remain until adulthood, increasing metabolic cardiovascular risk factors^10–13^. Bleil et al. observed in women aged 25–45 years that the association between metabolic cardiovascular risk factors [total cholesterol, high-density lipoprotein (HDL) cholesterol, low-density lipoprotein (LDL) cholesterol, triglycerides, fasting glucose, insulin and hypertension] and early age at menarche disappeared, after adjusting for body composition and waist circumference in adulthood^14^. On the other hand, other studies have failed to identify body composition as a mediator^15–17^. The association of age at menarche with markers of arterial stiffness^18,19^ and atherosclerosis^20,21^, such as pulse wave velocity and carotid intima-media thickness, has also been evaluated^7,22–24^.

The heterogeneous finding of the studies that evaluated whether body composition was a mediator of the association between age at menarche and metabolic cardiovascular risk factors may be due to the use of less precise techniques to measure body composition^14,16,25^. Furthermore, these studies neither estimate the indirect effect of body composition, nor adjusted the analysis to confounders of the association between mediator and outcome, nor for the interaction between age at menarche and mediator^26^. Thus, the use of mediation analysis techniques that consider the interaction between exposure and mediator, as well as, control for post-confounders^27^, such as G-computation^28^, overcome these limitations of previous used methods^29^.

Besides using standard measures of body composition (BMI, waist circumference and waist-to-hip ratio) and more precise methods, such as, fat mass index and abdominal visceral fat layer thickness, new studies should also estimate the indirect effect of age at menarche through body composition. Contributing to a better understanding of the pathways through early menarche affects the development of cardiometabolic diseases and whether there is a difference on the mediation among the body composition measures.

This study was aimed at assessing the association between age at menarche and metabolic cardiovascular risk factors (systolic blood pressure, diastolic blood pressure, carotid intima-media thickness, pulse wave velocity, total cholesterol, HDL-cholesterol, LDL-cholesterol, triglycerides, glucose, glycated hemoglobin, C-reactive protein). We also estimated the indirect effect of body composition at 30 years of age, among subjects who have been prospectively followed since birth in a southern Brazilian city.

## Results

In 2012–2013, 3701 subjects (men and women) were interviewed, which represented a follow-up rate of 68.1% of the original cohort, after taking into account the 325 deaths identified among the cohort members. Among women (mean age: 30.2 years) the follow-up rate of the original cohort was 71.1%. Of the 1914 women interviewed in 2012–2013, information on the age at menarche and metabolic cardiovascular risk factors was available for 1680. Women who were three months postpartum or pregnant (N: 69), with no limbs/amputations or metal in some part of the body, non-removable (N: 31), were excluded from the evaluations.

Table 1 presents the characteristics of the participants included in the present analysis. Most of the participants (70.7%) were born in families with a monthly income lower than 3 minimum wages and about one of every three mothers had 4 or less years of schooling (33.2%). The prevalence of low birth weight was 7.8%. The proportion reporting an age at menarche below 12 years of age was 24.5%.

**Table 1:** Characteristics of the study population.

Variables		N (%)/Mean (SD)	Variables		N (%)/Mean (SD)
**At birth**			**23 years**		
Household score index		3.32 (1.20)	Age at menarche (years)	< 12	411 (24.5)
Family income (minimum wages)	< 1	328 (19.6)		12–13	899 (53.5)
	1.1–3	854 (51.1)		≥ 14	370 (22.0)
	3.1–6	310 (18.6)	**30 years**		
	6.1–10	93 (5.6)	% of saturated fatty acid in the diet		10.12 (2.60)
	> 10	85 (5.1)	Leisure-time and commuting physical activity (minutes/week)	< 150	1320 (80.0)
Maternal schooling (years)	0–4	557 (33.2)		≥ 150	330 (20.0)
	5–8	709 (42.3)	Systolic blood pressure (mm Hg)		114.7 (11.9)
	9–11	185 (11.0)	Diastolic blood pressure (mm Hg)		74.0 (9.2)
	≥ 12	227 (13.5)	Carotid intima-media thickness (µm)		579.5 (15.9)
Maternal smoking	Yes	590 (35.1)	Pulse wave velocity (m/s)		6.39 (1.09)
	No	1090 (64.9)	Total cholesterol (mg/dl)		189.2 (35.7)
Birth weight (g)	< 2500	131 (7.8)	HDL-cholesterol (mg/dl)		63.3 (13.8)
	2500–2999	457 (27.2)	LDL-cholesterol (mg/dl)		106.7 (28.7)
	3000–3499	637 (37.9)	Triglycerides (mg/dl)^a^		90.2 (1.92)
	≥ 3500	454 (27.0)	Plasma glucose (mg/dl)		86.6 (20.5)
Genomic ancetry (%)	African	0.16 (0.19)	Glycated hemoglobin (%)		5.10 (0.50)
	European	0.77 (0.20)	C-reactive protein (mg/dl)^a^		1.30 (5.5)
	Native American	0.07 (0.05)	Body mass index (kg/m^2^)	< 25	766 (46.9)
**Childhood**				25–29.9	468 (28.7)
Breastfeeding (months)	< 1	323 (19.7)		≥ 30	399 (24.4)
	1–2.9	436 (26.7)	Abdominal visceral fat layer thickness (cm)		4.94 (1.68)
	3–5.9	382 (23.4)	Waist circumference (cm)		80.8 (12.1)
	6–8.9	153 (9.4)	Waist-to-hip ratio		76.1 (6.5)
	9–11.9	60 (3.7)	Fat mass index (kg/m^2^)		10.73 (4.75)
	≥ 12	282 (17.2)	Fat free mass index (kg/m^2^)		15.84 (1.87)

^a^Geometric mean and Interquartile range.

Table 2 reports the mean values in each strata of age at menarche (< 12; 12–13;  ≥ 14). Diastolic blood pressure, total cholesterol, LDL-cholesterol, triglycerides, waist-to-hip ratio, body mass index and fat mass index showed higher mean among women whose age at menarche was < 12 years in relation to those whose age at menarche was ≥ 14 years. These metabolic cardiovascular risk factors were inversely associated with age at menarche, and a linear trend was observed (Table 2).

**Table 2:** Mean and percentage of the metabolic cardiovascular risk factors at 30 years by age at menarche categories.

Metabolic cardiovascular risk factors	Age at menarche (years)—Mean/% (CI 95%)			*p* value
	< 12 (N: 411)	12–13 (N: 899)	≥ 14 (N: 370)	
Systolic blood pressure (mm Hg)	115.8 (114.6; 117.1)	114.2 (113.5; 114.9)	114.6 (113.2; 115.9)	0.07^b^
Diastolic blood pressure (mm Hg)	75.1 (74.2; 76.0)	73.8 (73.2; 74.4)	73.1 (72.2; 74.1)	0.003^a^
Total cholesterol (mg/dl)	194.7 (191.1; 198.3)	188.2 (185.9; 190.4)	185.5 (181.6; 189.4)	< 0.001^a^
HDL-cholesterol (mg/dl)	63.0 (61.7; 64.4)	63.5 (62.5; 64.4)	63.0 (61.6; 64.5)	0.81^b^
LDL-cholesterol (mg/dl)	110.9 (108.0; 113.8)	105.7 (103.9; 107.6)	104.2 (101.0; 107.3)	0.001^a^
Triglycerides (mg/dl)	111.8 (104.4; 119.1)	100.7 (97.0; 104.4)	96.1 (90.6; 101.6)	< 0.001^a^
C-reactive protein (mg/dl)	1.49 (1.20; 1.85)	1.27 (1.10; 1.47)	1.17 (0.91; 1.51)	0.31^b^
Waist-to-hip ratio	77.5 (76.6; 77.9)	76.1 (75.6; 76.5)	75.2 (74.6; 75.8)	< 0.0001^a^
Fat mass index (kg/m^2^)	12.54 (12.08; 13.01)	10.33 (10.06; 10.60)	9.72 (9.28; 10.16)	< 0.001^a^
BMI (kg/m^2^)	29.11 (28.5; 29.7)	26.21 (25.8; 26.6)	25.52 (24.9; 26.1)	< 0.001^a^

^a^Linear trend test.

^b^Heterogeneity test.

*p* value: difference in values between groups of age at menarche, *p* < 0.05 was statistically significant.

Table 3 shows that after controlling for confounding variables, diastolic blood pressure, total cholesterol, LDL-cholesterol, triglycerides, waist-to-hip ratio, body mass index and fat mass index remained associated with age at menarche (< 12 x ≥ 14), except for systolic blood pressure, HDL-cholesterol and C-reactive protein, because the confidence interval includes the null value.

**Table 3:** Estimation of metabolic cardiovascular risk factors at age 30 in relation to age at menarche categories.

Metabolic cardiovascular risk factors	Age at menarche (years)—regress coefficient (CI 95%)^c^			*p* value
	< 12 (N: 411)	12–13 (N: 899)	≥ 14 (N: 370)	
Systolic blood pressure (mm Hg)	1.25 (− 0.59; 3.11)	− 0.25 (− 1.84; 1.35)	Reference	0.15^b^
Diastolic blood pressure (mm Hg)	1.98 (0.56; 3.40)	0.67 (− 0.55; 1.89)	Reference	0.01^a^
Total cholesterol (mg/dl)	8.28 (2.67; 13.88)	1.83 (− 3.02; 6.67)	Reference	0.003^a^
HDL-cholesterol (mg/dl)	− 0.01 (− 2.10; 2.09)	0.47 (− 1.34; 2.28)	Reference	0.81^b^
LDL-cholesterol (mg/dl)	6.53 (2.00; 11.07)	1.26 (− 2.66; 5.18)	Reference	0.004^a^
Triglycerides (mg/dl)	0.11 (0.03; 0.19)	0.02 (− 0.05; 0.08)	Reference	0.003^a^
C-reactive protein (mg/dl)	0.10 (− 0.26; 0.45)	− 0.001 (− 0.31; 0.31)	Reference	0.78^b^
Waist-to-hip ratio	1.97 (0.97; 2.97)	0.98 (0.12; 1.84)	Reference	0.0001^a^
Fat mass index (kg/m^2^)	2.84 (2.16; 3.52)	0.51 (− 0.07; 1.10)	Reference	< 0.0001^a^
BMI (kg/m^2^)	3.58 (2.66; 4.50)	0.60 (− 0.2; 1.39)	Reference	< 0.0001^a^

^a^Linear trend test.

^b^Heterogeneity test.

^c^Adjusted for: family income, household score index, maternal schooling, maternal smoking, genomic ancestry, birthweight in grams and duration of breastfeeding.

*p* value: difference in values between groups of age at menarche, *p* < 0.05 was statistically significant. Regress coefficients represent the unit (media or proportion) increase in the metabolic risk factor, e.g., the mean diastolic blood pressure increases, on average, 1.98 mmHg, if age at menarche was < 12 relative to those with menarche age ≥ 14 years.

The Supplementary Table 1 presented analysis for the association of age at menarche with additional metabolic cardiovascular risk factors. Pulse wave velocity, waist circumference, fat free mass index, abdominal visceral fat layer thickness and overweight even after controlling for confounding remained association with early age at menarche.

Table 4 shows the results of the mediation analysis for each one of the body composition measures (BMI, fat mass index, waist-to-hip ratio) and the proportion mediated effect. Fat mass index captured most of the association of age at menarche with diastolic blood pressure (65.8%), carotid intima-media thickness (74.4%), pulse wave velocity (44.0%), LDL-cholesterol (98.9%), triglycerides (60.0%) and glycated hemoglobin (93.9%). The effect on systolic blood pressure, C-reactive protein and blood glucose was not mediated by fat mass index. BMI captured from 23.3 to 76.8% of the effect of early menarche with diastolic blood pressure, pulse wave velocity, total cholesterol, LDL-cholesterol and plasma glucose. These results are presented in last column of the Table 4. However, only diastolic blood pressure [fat mass index—NIE: 1.07 (0.24; 1.90)] and LDL-cholesterol [BMI—NIE: 3.50 (1.03; 5.97); waist-to-hip ratio—NIE: 2.66 (0.35; 4.97)] showed the evidence of mediation by body composition, because the confidence intervals did not include the null value. Thus, fat mass index captured 65.8% of the association of age at menarche with diastolic blood pressure and, BMI and waist-to-hip ratio captured 76.8% and 47.8%, respectively, of the association of age at menarche with LDL-cholesterol.

**Table 4:** Mediation analysis of the body composition on the association between age at menarche and metabolic cardiovascular risk factors at 30 years.

Metabolic cardiovascular risk factors	Mediator	Natural direct effect (NDE)	Natural indirect effect (NIE)	Total causal effect (TCE)	% Mediated effect
Systolic blood pressure (mmHg)^1^	BMI	− 0.78 (− 0.20; 0.63)	1.12 (0.08; 2.15)	0.33 (− 1.21; 189)	0
	Fat mass index	− 0.09 (− 1.55; 1.36)	1.33 (0.27; 2.38)	1.24 (− 0.40; 2.87)	0
	Waist-to-hip ratio	0.73 (− 0.59; 2.04)	0.43 (− 0.54; 1.40)	1.16 (− 0.21; 2.52)	37.1
Diastolic blood pressure (mmHg)^2^	BMI	0.58 (− 0.49; 1.65)	0.53 (− 0.26; 1.33)	1.11 (− 0.08; 2.30)	47.7
	Fat mass index	0.58 (− 0.55; 1.71)	1.07 (0.24; 1.90)	1.65 (0.37; 2.92)	65.8
	Waist-to-hip ratio	0.80 (− 0.22; 1.82)	0.01 (− 0.74; 0.75)	0.81 (− 024; 1.86)	1.2
Total cholesterol (mg/dl)^2^	BMI	1.71 (− 2.69; 6.12)	2.47 (− 0.55; 5.49)	4.18 (− 0.71; 9.06)	59.0
	Fat mass index	3.14 (− 1.36; 7.63)	2.45 (− 0.66; 5.58)	5.59 (0.56; 10.63)	43.8
	Waist-to-hip ratio	5.39 (1.23; 9.55)	− 1.36 (− 2.48; − 0.24)	3.39 (− 0.82; 7.59)	0
HDL-cholesterol (mg/dl)^1^	BMI	0.84 (− 0.81; 2.50)	− 0.42 (− 1.52; 0.68)	0.42 (− 1.40; 2.25)	0
	Fat mass index	1.16 (− 0.58; 2.90)	− 0.56 (− 1.15; 1.97)	0.61 (− 1.32; 2.53)	0
	Waist-to-hip ratio	0.02 (− 1.52; 1.57)	0.19 (− 0.91; 1.30)	0.22 (− 1.42; 1.85)	86.4
LDL-cholesterol (mg/dl)^2^	BMI	1.06 (− 2.42; 4.55)	3.50 (1.03; 5.97)	4.56 (0.68; 8.44)	76.8
	Fat mass index	0.04 (− 3.53; 3.61)	3.74 (1.21; 6.27)	3.78 (− 0.25; 7.81)	98.9
	Waist-to-hip ratio	2.91 (− 0.40; 6.22)	2.66 (0.35; 4.97)	5.57 (2.18; 8.96)	47.8
Triglycerides (mg/dl)a,^1^	BMI	− 0.004 (− 0.06; 0.05)	0.04 (0.004; 0.09)	0.04 (− 0.02; 0.11)	0
	Fat mass index	0.02 (− 0.04; 0.07)	0.03 (− 0.01; 0.08)	0.05 (− 0.02; 0.11)	60
	Waist-to-hip ratio	0.04 (− 0.013; 0.10)	0.01 (− 0.03; 0.04)	0.05 (− 0.01; 0.10)	20
C-reactive-protein (mg/dl)a,^1^	BMI	− 0.12 (− 0.26; 0.02)	0.20 (0.10; 0.30)	0.08 (− 0.09; 0.25)	0
	Fat mass index	− 0.09 (− 0.24; 0.06)	0.19 (0.09; 0.30)	0.10 (− 0.08; 0.28)	0
	Waist-to-hip ratio	0.12 (− 013; 025)	0.03 (− 0.06; 0.12)	0.15 (0.01; 0.29)	20

^a^Logaritmo adjusted multiple regress.

^1^*p *value > 5%.

^2^*p* value ≤ 5%.

TCE: NDE + NIE, total effect of the association between age at menarche and metabolic cardiovascular risk factors; NIE: effect of the association between age at menarche and metabolic cardiovascular risk factors through mediator; NDE: represent the effect of the association between age at menarche and metabolic cardiovascular risk factors controlled by mediator; e.g., TCE: the mean diastolic blood pressure increases, on average, 1.11 mmHg in women with menarche < 12 years, NIE: the mean diastolic blood pressure increases, on average, 0.53 mmHg when the effect through mediator BMI, NIE: the mean diastolic blood pressure increases, on average, 0.58 mmHg when the effect was controlled by mediator BMI. When the direct and indirect effect occurred in opposite directions, the mediated % is reported to be 0.

## Discussion

Among young adult women, age at menarche < 12 years was associated with higher levels of metabolic cardiovascular risk factors. The mediation analysis showed that body composition measured by fat mass index, BMI and waist-to-hip ratio at 30 years old, captured part of the effect of age at menarche on diastolic blood pressure and LDL-cholesterol. In spite of the fact that most of the confidence intervals of the indirect effect included the unit, our analysis suggests that body composition in adulthood is an important mechanism in the association of early menarche with increased cardiovascular risk. Although the follow-up rates at 30 years were slightly higher among individuals in the intermediate income categories, these differences were small, at most 10% points^31^. Therefore, it is unlikely that the observed associations were due to selection bias.

Regarding the mediation analysis, unlike the previously published studies, we estimated the indirect effect of body composition, which allowed the assessment of the proportion of the effect of early menarche that was captured by body composition (BMI, fat mass index and waist-to-hip ratio). In addition, this analysis controlled for confounding factors in the mediator-outcome association and for interaction between exposure and mediator^27,29,38^. Another strength of this study is the use of more accurate methods to evaluate body composition, that are able to disentangle lean from fat mass^39,40^.

The length of recall on age at menarche could be considered as a limitation of our study, on average, 10.6 years. Such long recall time could have caused an error in women's reporting, introducing a non-differential misclassification error. Because non-differential misclassification tends to underestimate the magnitude of the association, the observed associations are unlikely to be due to recall bias.

Measurement error can lead to large biases in mediation analysis, but it is believed that such error did not occur in our study due to the rigor in obtaining the measurements and performing the exams. A protocol was developed for each of the measures, with the training of the team of professionals, who carried out the evaluations. All anthropometric measurements were performed twice and when the difference between the two measurements was above the acceptable error, a third one was performed. Body composition assessments were carried out with special clothing to reduce measurement error. Daily calibrations and weekly quality control were executed for DEXA.

Similarly, to studies carried out in different socioeconomic contexts, we observed that early age at menarche is associated with higher diastolic blood pressure and triglycerides among young adult women^41^. Some studies have shown an intimate relationship between obesity/overweight, assessed by BMI, with diastolic function^42–44^, as well as the association between age at menarche and BMI-mediated diastolic blood pressure^23,45^. The use of more accurate measures of body composition to quantify the accumulation of body fat, contribute better to the prevention and intervention in cardiometabolic problems. Our study shows this, when it identified that a higher percentage of mediation of the association between age at menarche and diastolic blood pressure was through the fat mass index (65.8%) than BMI (47.7%).

On the other hand, we did not observe an association between age at menarche and other metabolic cardiovascular risk factors, such as HDL-cholesterol and C-reactive protein, unlike other studies^6,16,17,46^. This may have occurred because we evaluated women at younger age (30 years) than the previous studies.

In another study, we observed that the association between age at menarche and body composition in adulthood was mainly related to body composition in late childhood^47^. The difference in fat mass index, comparing those whose age at menarche was < 12 years, in relation to 14 years or more, reduced from 2.2 kg/m^2^ (95% CI: 1.7; 2.6) to 0.26 (95% CI: − 0.008; 0,60), after adjustment for BMI z-score at 11 years.

In this study, we did not have information about pre-pubertal body composition. However, if we had this variable and adjusted in the analysis of the association between age at menarche and metabolic cardiovascular risk factors, the effect mediated by adult body composition would be reduced, hiding an important explanatory factor of the association. Therefore, the absence of the information on pre-pubertal body composition did not bias the analyzes performed in this study, since adult body composition accounted for most of the relationship between age at menarche and metabolic cardiovascular risk factors^23^. Despite the fact that measures of body composition and metabolic cardiovascular risk factors were evaluated at the same time, which could cause doubts as to the validity of the associations, violating the principle of temporality, we do not believe that this could have occurred in this study. First, because the age of menarche < 12 years is a marker of excess body fat^47^, this excess weight being taken into adulthood^48^, which can cause cardiometabolic problems later on. Second, in mediation analyses, we controlled for confounders of the mediator-outcome association, reducing the possibility that the effect estimate was biased. Finally, all body composition measures were performed by different professionals and they did not have access to the results of the other exams, reducing the tendency to measurement error.

Among the body composition measures, fat mass index and waist-to-hip ratio captured most of the association between age at menarche and metabolic cardiovascular risk factors^23,45^, evidencing the importance of obtaining more precise and various kinds of measures of body composition. In the analyses of this article, we sought to assess the contribution of each of the measures of body composition in the association of age at menarche and metabolic cardiovascular risk factors. BMI and waist-to-hip ratio are widely used in epidemiological studies and in the daily routine of health services, as they are easy to obtain and interpret. The fat mass index is a non-invasive exam that accurately indicate the amount of body fat and its location, however, with restricted access due to the high cost and need for a specialized professional. For this reason, each of these body composition measurements was tested to estimate the proportion of the associations between age at menarche and metabolic cardiovascular risk factors was mediated by each of them. Thus, it was observed that among the body composition measures, fat mass index mediated in greater proportion a higher number of associations (diastolic blood pressure, LDL-cholesterol, triglycerides), than waist-to-hip ratio (systolic blood pressure, HDL-cholesterol, C-reactive protein) and BMI (total cholesterol). For the four tested association (systolic blood pressure, HDL-cholesterol, triglycerides and C-reactive protein), BMI mediation was zero. This analysis reinforces that BMI should not be used as the only indicator of cardiometabolic risk among young adult women. Measure such as waist-to-hip ratio should be assessed simultaneously with BMI, and when available fat mass index should be estimated.

Although in the mediation analysis (Table 4) most NIE confidence intervals include the null value, we believe that adult body composition plays an important role in mediating the tested associations between age at menarche and metabolic cardiovascular risk factors. Mendelian randomization, which uses genetic variants as a proxy for the exposure of interest, has been an important analysis technique in observational studies^49^, as it reduces the possibility of interference from confounding factors in the tested associations, between exposure and outcome measured in the population. Studies using Mendelian randomization have found that adiposity mediates most of the association between age at menarche and metabolic cardiovascular risk factors^50^, and that younger age at menarche has a causal effect of increasing adult BMI, even after excluding variants with evidence of horizontal BMI pleiotropy in childhood^51^.

In a public health context, this study shows the importance of preventing overweight/obesity mainly among women who had early menarche, because this is a marker of body composition in childhood.

## Methods

In 1982, maternity hospitals in Pelotas, a southern Brazilian city, were visited daily and all births were identified. Live births (N = 5914; 2876 women) whose family lived in the urban area of the city, were examined and their mothers interviewed shortly after birth. These individuals have been followed for several times. Further information on the study methodology has been published elsewhere^30,31^.

Between June 2012 and February 2013, we tried to follow the whole cohort, and participants were invited to attend the research clinic, where they were interviewed, examined and a blood sample was collected^31^.

In 2012–2013 visit (N: 1914 women), women who were pregnant or in the three months postpartum period (N: 69) and those that had no limbs or that had metal in some part of the body (N: 31) were excluded from physical and/or blood exams.

Pulse wave velocity was assessed for women in the three-month postpartum period, and only pregnant women were excluded from this assessment. In the data analysis, 1680 female members of the Cohort who had information on at least one metabolic cardiovascular risk factor and age at menarche were included, which added to 130 deaths represented the follow-up proportion of 62.9% of the original cohort.

- Exposure variable

Information on age at menarche was gathered in the 23 years follow-up visit (N: 2083 women), women were asked about the age at first menstrual period.

- Cardiometabolic outcomes

Blood pressure was evaluated with an automatic arm cuff monitor, model HEM-705CPINT, Omron, for obese individuals a specific cuff was used. The measurement was performed twice (minimum interval of 2 min), after 5 min of rest, with the individual sitting, uncrossed legs, left arm supported at the height of the heart. In the analysis, the mean of the two measures of systolic and diastolic blood pressure was used.

Pulse wave velocity was assessed using a portable ultrasound, SphygmoCor System (AtCor Medical, Version 9.0, Sydney, Australia), after 5 min of rest, with the subject lying in supine position. Pulse wave velocity was estimated by dividing the distance between the two measurement sites (carotid and femoral) by the time of transit between the femoral and carotid pulse, expressed in meters per second (m/s).

Intima-media thickness of the right and left carotid was evaluated using a Toshiba Model Xario Ultrasound, with the person lying in the supine position and the head lateralized that was measured at the posterior wall of the right and left common carotid arteries in longitudinal planes. Measurement were obtained using the Carotid Analyzer for Research, Medical Imaging Application (MIA-LLC) software (Carotid Analyser, Iowa City, Iowa), it automatically calculated the mean value of 90 measurements (frames) taken in the 10-mm-long section studied. In the analysis, we used the arithmetic mean of the measurements, expressed in micrometers (μm)^21^.

Total cholesterol, HDL-cholesterol, LDL-cholesterol, triglycerides and plasma glucose were assessed in a BS-380, Mindray, by automated colorimetric enzymatic method, whereas the C-reactive protein was measured by the automated turbidimetry technique. The % of glycated hemoglobin was estimated by high performance liquid chromatography, associated with ionic exchange chromo-chromatography, in Bio-Rad brand apparatus.

- Anthropometry

Weight was evaluated using a scale with 150 kg capacity coupled to the Bod Pod equipment and height by a portable stadiometer with a precision of 0.1 cm. The presence of overweight was defined by body mass index (BMI) ≥ 25 kg/m^2^. Waist circumference was measured with the subject standing, arms relaxed at the side of the body and feet together, twice. Hip circumference was measured horizontally in the most protruding points back of the gluteal region (anteroposterior and lateral planes) with the subject wearing light clothes. When these measures had a discrepancy greater than 1 cm, a third assessment was performed, and the means of the measures was used in the analysis. Waist-to-hip ratio was determined by dividing the circumference of the waist by of the hip.

- Body composition

Fat mass and fat free mass were evaluated using Dual-energy X-ray Absorptiometry (DXA) model Lunar Prodigy, brand GE Healthcare. Pregnant women or suspected of pregnancy did not take part in this assessment. Fat and fat free mass indexes were obtained by dividing fat and fat free measurements by the height in meters squared. Visceral fat layer thickness (cm) was estimated with the subject lying in the supine position, using Toshiba Model Xario Ultrasound with a 3.5-MHz convex probe.

- Confounders

Family income in minimum wages, household score index (estimated through factor analysis and based in the ownership of household goods), maternal schooling (years), maternal smoking (yes or not) and birthweight in grams (assessed by hospital staff using pediatric scales that were calibrated weekly by the research team), were evaluated in the perinatal study^32^. Duration of breastfeeding, reported by the mother at the two-year visit, assessed the age, in months, at which breastfeeding was completely stopped. Genomic ancestry was estimated in the 23-year follow-up based on approximately 370,000 single nucleotide polymorphisms (SNPs) available in the Pelotas cohort and HapMap and the Human Genome Diversity Project, and the ADMIXTURE program estimated the proportion of European, African-American and Native American ancestry^33^. Only African ancestry was used for adjustment, since the Native American had low frequency and the European and African were highly correlated (Pearson correlation coefficient: − 0.97).

- Analyses

**Figure 1:**
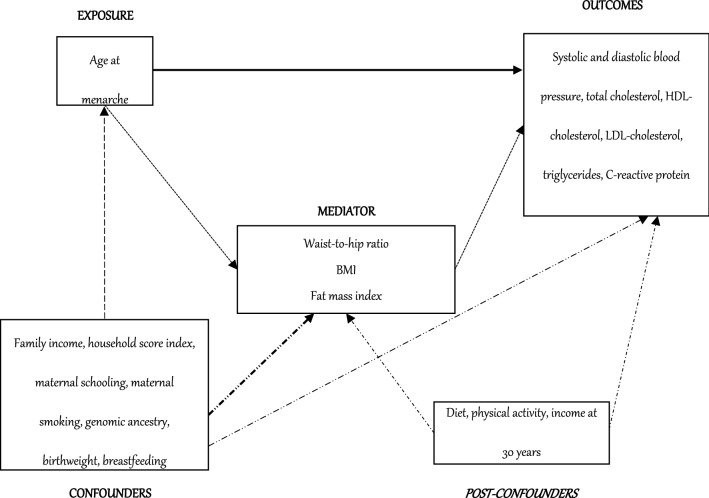
Directed acyclic chart (DAG) representing the influence of mediator and confounding variables in the relationship between age at menarche and metabolic cardiovascular risk factors.

Data analysis was performed using Stata software version 12.1 (Stata Corp, College Station, TX, USA). C-reactive protein and triglycerides were log-transformed because of their asymmetric distribution. Analysis of variance (ANOVA) was used to compare means. Multiple linear regression was carried out to each of the metabolic cardiovascular risk factors, separately, adjusting the estimates for confounders (family income, household score index, maternal schooling, maternal smoking, genomic ancestry, birthweight and duration of breastfeeding). In the analysis of C-reactive protein, measures > 10 mg/dl (indicates acute inflammation), pregnant women, in postpartum (≤ 3 months) and using oral contraceptive were excluded^34,35^. Comparison of proportions among categories of age at menarche was performed using chi square test and prevalence ratio was estimated using Poisson regression with robust adjustment of the variance. Mediation analysis used G-computation (bootstrap replications: 10,000) to estimate the direct effect of age at menarche and the indirect effect of waist-to-hip ratio, BMI and fat mass index, separately, for each of the metabolic cardiovascular risk factors. In this analysis, the following variables were considered as post-confounders: leisure-time and commuting physical activity (≥ 150 min/week: active; < 150: non-active) and % of saturated fats in the diet (saturated fats were obtained by adjusted for total caloric value), at the 30-year follow-up^36^. Family income, household score index, maternal schooling, maternal smoking, genomic ancestry, birthweight and duration of breastfeeding were considered as base confounders. This analysis also adjusted the estimates for the interaction between age at menarche and mediator (Fig. 1). The mediation analysis used G-computation to estimate the total causal effect (TCE)—represent the total effect of the association between age at menarche and metabolic cardiovascular risk factors, natural indirect effect (NIE)—represent the effect of the association between age at menarche and metabolic cardiovascular risk factors through a mediator, natural direct effect (NDE)—represent the effect of the association between age at menarche and metabolic cardiovascular risk factors controlled by mediator. Thus, TCE is the sum of the NDE and NIE. Proportion of the mediated effect was obtained using the formula: (NIE/TCE) * 100^37^.

The study was approved by the Research Ethics Committee of Federal University of Pelotas, Rio Grande do Sul, Brazil (Number: 16/2012) and performed in accordance with ethical guidelines. Written informed consent was obtained from all participants and/or their legal guardians.

## Supplementary Information

Supplementary information.

## Data Availability

The datasets generated during and/or analysed during the current study are available from the corresponding author on reasonable request. The Stata code used for this analysis is available at https://github.com/sbubach/Code-Data-manuscript-SR with DOI: https://zenodo.org/badge/latestdoi/258943392.
