# Supplementary material for: Early age at menarche and metabolic cardiovascular risk factors: mediation by body composition in adulthood
Source: Sci Rep. 2021 Jan 8;11:148. doi: 10.1038/s41598-020-80496-7 (PMC7794383; doi:10.1038/s41598-020-80496-7)
Supplement: Supplementary file 1 — Supplementary information. [file 41598_2020_80496_MOESM1_ESM.docx]

**“Early age at menarche and metabolic cardiovascular risk factors: mediation by body composition in adulthood”**

**Susana Bubach, Bernardo Lessa Horta, Helen Gonçalves, and Maria Cecília Formoso Assunção.**

**Supplementary material**

**Supplementary table 1.** Estimation of metabolic cardiovascular risk factors at age 30 in relation to age at menarche categories.

| **Metabolic cardiovascular risk factors** | **Age at menarche (years) – Regress coefficient**  **(CI 95%)**^c^ | | | **P-value** |
| --- | --- | --- | --- | --- |
|  | **<12 (N: 411)** | **12-13 (N: 899)** | **≥14 (N: 370)** |  |
| **Carotid intima-media thickness (µm)** | 1.33 (-1.33; 4.00) | -0.59 (-2.90; 1.71) | Reference | 0.22^b^ |
| **Pulse wave velocity (m/s)** | 0.39 (0.12; 0.66) | 0.22 (-0.02; 0.45) | Reference | 0.01^a^ |
| **Plasma glucose (mg/dl)** | 1.81 (-1.30; 4.93) | 1.81 (-0.88; 4.50) | Reference | 0.39^b^ |
| **Glycated hemoglobin (%)** | 0.05 (-0.02; 0.13) | 0.03 (-0.03; 0.10) | Reference | 0.39^b^ |
| **Waist circumference (cm)** | 6.51 (4.66; 8.36) | 1.43 (-0.17; 3.02) | Reference | <0.0001^a^ |
| **Abdominal visceral fat layer thickness (cm)** | 0.52 (0.26; 0.78) | 0.11 (-0.11; 0.33) | Reference | 0.0001^a^ |
| **Fat free mass index (kg/m²)** | 0.76 (0.47; 1.05) | 0.08 (-0.16; 0.33) | Reference | <0.0001^a^ |
| **Overweight (%)** | 1.59 (1.38; 1.83) | 1.12 (0.97; 1.29) | Reference | <0.0001^a^ |

^a^Linear trend test, ^b^Heterogeneity test, ^c^Adjusted for: family income, household score index, maternal schooling, maternal smoking, genomic ancestry, birthweight in grams and duration of breastfeeding.
